# The quantity and quality of complementary and alternative medicine clinical practice guidelines on herbal medicines, acupuncture and spinal manipulation: systematic review and assessment using AGREE II

**DOI:** 10.1186/s12906-016-1410-8

**Published:** 2016-10-29

**Authors:** Jeremy Y. Ng, Laurel Liang, Anna R. Gagliardi

**Affiliations:** Toronto General Hospital Research Institute, University Health Network, Toronto, Ontario Canada

**Keywords:** Complementary and alternative medicine, Integrative medicine, Systematic review, AGREE II, Clinical practice guideline

## Abstract

**Background:**

Complementary and alternative medicine (CAM) use is often not disclosed by patients, and can be unfamiliar to health care professionals. This may lead to underuse of beneficial CAM therapies, and overuse of other CAM therapies with little proven benefit or known contraindications. No prior research has thoroughly evaluated the credibility of knowledge-based resources. The purpose of this research was to assess the quantity and quality of CAM guidelines.

**Methods:**

A systematic review was conducted to identify CAM guidelines. MEDLINE, EMBASE and CINAHL were searched in January 2016 from 2003 to 2015. The National Guideline Clearinghouse, National Center for Complementary and Integrative Health web site, and two CAM journals were also searched. Eligible guidelines published in English language by non-profit agencies on herbal medicine, acupuncture, or spinal manipulation for adults with any condition were assessed with the Appraisal of Guidelines, Research and Evaluation II (AGREE II) instrument.

**Results:**

From 3,126 unique search results, 17 guidelines (two herbal medicine, three acupuncture, four spinal manipulation, eight mixed CAM therapies) published in 2003 or later and relevant to several clinical conditions were eligible. Scaled domain percentages from highest to lowest were clarity of presentation (85.3 %), scope and purpose (83.3 %), rigour of development (61.2 %), editorial independence (60.1 %), stakeholder involvement (52.0 %) and applicability (20.7 %). Quality varied within and across guidelines. None of the 17 guidelines were recommended by both appraisers; 14 were recommended as Yes or Yes with modifications.

**Conclusions:**

Guidelines that scored well could be used by patients and health care professionals as the basis for discussion about the use of these CAM therapies. In future updates, guidelines that achieved variable or lower scores could be improved according to specifications in the AGREE II instrument, and with insight from a large number of resources that are available to support guideline development and implementation. Future research should identify CAM therapies other than those reviewed here for which guidelines are available. Research is also needed on the safety and effectiveness of CAM therapies.

**Electronic supplementary material:**

The online version of this article (doi:10.1186/s12906-016-1410-8) contains supplementary material, which is available to authorized users.

## Background

It is currently estimated that more than 70 % of North Americans have tried at least one form of complementary and alternative medicine (CAM), [1–3] collectively spending billions of dollars annually on these therapies [4, 5]. CAM has been defined as “a group of diverse medical and health care interventions, practices, products or disciplines that are not generally considered part of conventional medicine” [6]. The National Center for Complementary and Integrative Health (NCCIH) further defines a non-mainstream practice used *together with* conventional medicine as “complementary”, a non-mainstream practice used *in place of* conventional medicine as “alternative”, and the coordinated delivery or use of conventional and complementary approaches as “integrative” [6]. This study henceforth refers to therapies that fall into all of these categories as CAM.

The past several decades have seen a sharp increase in research on CAM given the strong patient-driven market [7]. Examples of well-studied CAM therapies that show potential benefit include chiropractic spinal manipulation for low back pain and headaches [8–12], and acupuncture for different types of pain [13–18]. Recognizing such benefits, academic institutions are increasingly incorporating CAM into medical education, research and practice [11]. However, a variety of factors appear to influence whether and how CAM is used. Patients may not discuss their use of CAM with health care professionals out of fear of being judged or not seeing this as important to disclose, potentially leading to contraindications with other treatment [19–22]. Many health care professionals were not exposed to CAM in their medical training [23], are unfamiliar with CAM therapies, and find it challenging to discuss use or disuse of CAM with their patients [24, 25]. This is exacerbated by the fact that CAM is comprised of many different and unrelated types of therapies and schools of thought about their use [26]; and the reliability of evidence about safety and effectiveness varies between CAM therapies [27–29]. Given all of these factors, concerns have been raised about legal and ethical issues pertaining to the recommendations that health care professionals offer their patients about using or not using CAM therapies [24, 30]. Hence, patients and health care professionals may benefit from credible, knowledge-based resources upon which to base discussions and decisions about use of CAM.

Health care professionals often rely on evidence-informed clinical practice guidelines to understand whether use of a given therapy is recommended, and as a basis for informed and shared decision-making with patients about associated risks and benefits [31]. Research on a variety of clinical topics has identified that overuse, underuse or misuse of therapies may be associated with guidelines that are of poor quality [32], and the quality of guidelines has been proven to vary considerably [33]. Few studies have examined CAM guidelines. Content analysis of 10 guidelines on cardiovascular disease and type II diabetes revealed that CAM-relevant information was brief, in some cases unclear, inconclusive and lacking in direction for health care professionals [34]. Analysis of 65 National Institute for Health and Clinical Excellence guidelines available in 2009 found that, among 17 guidelines that mentioned CAM, it was not clinically relevant to most; in 14 of 48 guidelines that did not mention CAM, available evidence on the safety and effectiveness of relevant CAM therapies had not been included [35]. Therefore, no research has thoroughly evaluated the credibility of CAM guidelines. An understanding of the nature of CAM guidelines available to support informed and shared decision-making among patients and providers would help to identify whether such resources are absent and thus needed, or how they could be improved, thereby guiding future guideline development and associated research. The purpose of this study was to assess the quantity and quality of CAM guidelines.

## Methods

### Approach

A systematic review was conducted to identify CAM guidelines using standard methods [36] and Preferred Reporting Items for Systematic Reviews and Meta-Analyses (PRISMA) criteria [37]. A protocol was not registered. Eligible guidelines were assessed with the widely used and validated Appraisal of Guidelines, Research and Evaluation II (AGREE II) instrument [38]. AGREE II is a tool that assesses the methodological rigour and transparency in which a guideline is developed, and is the international “gold standard” for the assessment of guidelines. Detailed information is available on the AGREE web site [www.agreetrust.org]. It consists of 23 items grouped in six domains: scope and purpose, stakeholder involvement, rigor of development, clarity and presentation, applicability, and editorial independence.

### Eligibility criteria

Eligibility criteria for CAM guidelines were based on the Population, Intervention, Comparison and Outcomes framework. Eligible *populations* were adults aged 19 years and older with any diseases or conditions. With respect to *interventions*, guidelines were more likely to have been published on CAM interventions for which evidence has accumulated. We referred to a bibliometric and content analysis of CAM trials in the Cochrane Library by Wieland et al. [39] which found that the CAM therapies most commonly evaluated in trials included herbal supplements (non-vitamin, non-mineral dietary supplements or Chinese herbal medicine), acupuncture, and chiropractic or osteopathic manipulation [39]. For this study, guidelines were eligible if they specifically focused on any of these CAM therapies (category 1 – CAM-specific), or were general CAM guidelines that included at least one recommendation (for or against) at least of these CAM therapies (category 2 – CAM-general). We excluded general guidelines, which includes many hundreds and perhaps thousands, as it would have been challenging to search for and screen them for potential mention of CAM. *Comparisons* pertained to the assessed quality of CAM guidelines. *Outcomes* were AGREE II scores which reflect guideline content and format. The following conditions were also applied to define eligible guidelines: developed by non-profit organizations including academic institutions, government agencies, disease-specific foundations, or professional associations or societies; published in 2003 or later, which corresponds to the publication of AGREE II which provides developers with criteria for developing high-quality guidelines; English language; and either publicly available or could be ordered through our library system. Publications in the form of consensus statements, protocols, abstracts, conference proceedings, letters or editorials; based on primary studies that evaluated CAM therapies; or focused on CAM curriculum, education, training, research, professional certification or performance were not eligible.

### Searching and screening

MEDLINE, EMBASE and CINAHL were searched on January 28, 2016 from 2003 to 2015 inclusive. The search strategy (Additional file 1) included Medical Subject Headings and keywords that reflect terms commonly used in the literature to refer to CAM [7]. We also searched the National Guideline Clearinghouse, a publicly available repository of guidelines [http://www.guideline.gov/] using keyword searches restricted based on the eligibility criteria including “acupressure”, “acupuncture”, “Chinese medicine”, “chiropractic”, “chiropractor”, “herbal medicine”, “herbal supplement”, “herbal therapy”, “osteopath”, “phytotherapy”, “plant extract” and “spinal manipulation”. Next, we searched the NCCIH web site which contained a single list of CAM guidelines [https://nccih.nih.gov/health/providers/clinicalpractice.htm]; and the tables of contents of two CAM journals with the highest impact factors: *BMC Complementary and Alternative Medicine* [https://bmccomplementalternmed.biomedcentral.com/] and the *Journal of Complementary and Alternative Medicine* [http://www.liebertpub.com/overview/journal-of-alternative-and-complementary-medicine-the/26/] from January 2011 and December 2015. All three authors independently screened the titles and abstracts recovered from MEDLINE to standardize screening by discussing and resolving selection differences. Following this, JYN and LL screened titles and abstracts from all other sources. JYN and ARG screened full-text items to confirm eligibility.

### Data extraction and analysis

The following data were extracted from each guideline and summarized: date of publication, country of first author; type of organization that published the guideline (academic institutions, government agencies, disease-specific foundations, or professional associations or societies); topic category 1 (CAM-specific) or category 2 (CAM-general); and guideline topic including type of CAM therapy and disease or condition. Most data were available in the guideline; to assess applicability, the web site of each developer was browsed and searched for any associated knowledge-based resources in support of implementation.

### Guideline quality assessment

The extraction and analysis of data from eligible guidelines followed standardized methods for applying the AGREE II instrument [38]. To do this we used the instructional manual provided by AGREE for this purpose. This is a 60-page document that first describes the AGREE instrument, provides instructions on how to apply the instrument then, for each domain, provides detailed guidance on where to look in the guideline for relevant content to judge that domain and how to rate each item in that domain. First a pilot test of the AGREE II instrument was conducted with two guidelines during which all three authors independently assessed both guidelines with the AGREE II instrument. Discrepancies were discussed and resolved. JYN and LL then independently assessed all eligible guidelines for 23 items across six domains using a seven-point Likert scale from strongly disagree (1) to strongly agree (7) that the item is met; rated the overall quality of each guideline (1 to 7); and used that information to recommend for or against use of each guideline. ARG resolved differences. Average appraisal scores were calculated by taking the average rating for all 23 items of a single appraiser of a single guideline, followed by taking the average of this value for both appraisers. Average overall assessments were calculated as the average of both appraisers’ “overall guideline assessment” scores for each guideline. Scaled domain percentages were generated for inter-domain comparison, and were calculated by adding both appraisers’ ratings of items within each domain, and scaling by maximum and minimum possible domain scores, before converting this into a percentage. Average appraisal scores, average overall assessments and scaled domain percentages for each guideline was tabulated for comparison.

## Results

### Search results (Fig. 1)

Searches retrieved 3,350 items, 3,126 were unique, and 3,095 titles and abstracts were eliminated, leaving 31 full-text guidelines that were considered. Of those, 14 were not eligible, primarily because they were not focused on CAM (7), they could not be retrieved (3), or did not meet other eligibility criteria (4), leaving 17 guidelines eligible for review.

**Fig. 1:**
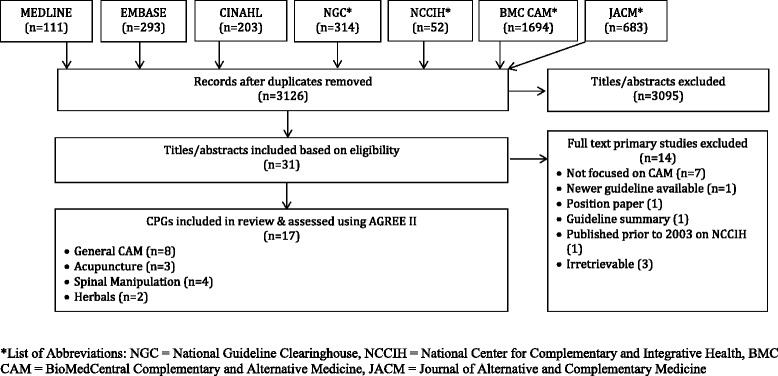
PRISMA diagram

### Guideline characteristics (Table 1)

Eligible guidelines were published in 2003 or later in Canada, the United States, United Kingdom, China, and Australia [40–56]. The guidelines were funded and/or developed by professional associations or societies (13), academic (3), and an international agency (1). Nine guidelines were CAM-specific (2 herbal medicine, three acupuncture, four spinal manipulation) and 8 were CAM-general. Clinical topics included anorexia nervosa, breast cancer, cancer (general) diabetes, headache, herpes zoster, low back pain, lung cancer, major depressive disorder, migraine, multiple sclerosis, neck pain, and Parkinson’s disease.

**Table 1:** Characteristics of eligible guidelines

Guideline	Country (First Author)	Developer	CAM category	Guideline topic
Fogarty 2015 [40]	Australia	Unclear	Acupuncture	Acupuncture for Anorexia Nervosa
Bryans 2014 [41]	Canada	Canadian Chiropractic Association	Spinal manipulation	Chiropractic Treatment for Neck Pain
Greenlee 2014 [42]	United States	Society for Integrative Oncology	General CAM	Integrative Therapies as Supportive Care in Breast Cancer Patients
Yadav 2014 [43]	United States	American Academy of Neurology	General CAM	Complementary and Alternative Medicine in Multiple Sclerosis
Deng 2013 [44]	United States	American College of Chest Physicians	General CAM	Complementary Therapies and Integrative Medicine in Lung Cancer
Liu 2013 [45]	China	Unclear; Sponsored by World Health Organization	Acupuncture	Acupuncture for Herpes Zoster
Nahas 2013 [46]	Canada	Canadian Diabetes Association	Herbals	Natural Health Products for Diabetes
Holland 2012 [47]	United States	American Academy of Neurology	General CAM	NSAIDs and Complementary Treatments for Episodic Migraine Prevention
Bryans 2011 [48]	Canada	Canadian Chiropractic Association	Spinal manipulation	Chiropractic Treatment of Headache
Seffinger 2010 [49]	United States	American Osteopathic Association	Spinal manipulation	Osteopathic Manipulative Treatment for Low Back Pain
Deng 2009 [50]	United States	Society for Integrative Oncology	General CAM	Complementary Therapies and Botanicals for Integrative Oncology
Ravindran 2009 [51]	Canada	Canadian Psychiatric Association, Canadian Network for Mood and Anxiety Treatments	General CAM	Complementary and Alternative Medicine for the Management of Major Depressive Disorder
Filshie 2006 [52]	United Kingdom	Unclear	Acupuncture	Providing Acupuncture for Cancer Patients
Suchowersky 2006 [53]	Canada	American Academy of Neurology	General CAM	Neuroprotective Strategies and Alternative Therapies for Parkinson Disease
Anderson-Peacock 2005 [54]	Canada	Canadian Chiropractic Association, Canadian Federation of Chiropractic Regulatory Boards	Spinal manipulation	Chiropractic Treatment for Neck Pain
Werneke 2005 [55]	United Kingdom	Unclear	General CAM	Complementary Therapies for Cancer
Mechanick 2003 [56]	United States	American Association of Clinical Endocrinologists	Herbals	Clinical Use of Dietary Supplements and Nutraceuticals

**Table 2:** Overall recommendations for use of appraised guidelines

Guideline	Appraiser 1	Appraiser 2
Fogarty 2015 [40]	Yes with Modifications	Yes with Modifications
Bryans 2014 [41]	Yes with Modifications	Yes with Modifications
Greenlee 2014 [42]	Yes with Modifications	Yes with Modifications
Yadav 2014 [43]	Yes with Modifications	Yes with Modifications
Deng 2013 [44]	No	Yes with Modifications
Liu 2013 [45]	No	Yes with Modifications
Nahas 2013 [46]	Yes with Modifications	Yes
Holland 2012 [47]	Yes with Modifications	Yes with Modifications
Bryans 2011 [48]	Yes with Modifications	Yes with Modifications
Seffinger 2010 [49]	Yes with Modifications	Yes with Modifications
Deng 2009 [50]	No	Yes with Modifications
Ravindran 2009 [51]	Yes with Modifications	Yes with Modifications
Filshie 2006 [52]	No	No
Suchowersky 2006 [53]	Yes with Modifications	Yes with Modifications
Anderson-Peacock 2005 [54]	Yes with Modifications	Yes with Modifications
Werneke 2005 [55]	No	No
Mechanick 2003 [56]	Yes with Modifications	Yes with Modifications

**Table 3:** Scaled domain percentages for appraisers of each guideline

Guideline	Domain score (%)					
	Scope and purpose	Stakeholder involvement	Rigour of development	Clarity of presentation	Applicability	Editorial Independence
Fogarty 2015 [40]	94.4	47.2	65.6	75.0	94.4	47.2
Bryans 2014 [41]	88.9	47.2	75.0	80.6	88.9	47.2
Greenlee 2014 [42]	100.0	72.2	80.2	97.2	100.0	72.2
Yadav 2014 [43]	97.2	47.2	77.1	77.8	97.2	47.2
Deng 2013 [44]	83.3	58.3	60.4	91.7	83.3	58.3
Liu 2013 [45]	52.8	11.1	32.3	88.9	52.8	11.1
Nahas 2013 [46]	75.0	86.1	92.7	91.7	75.0	86.1
Holland 2012 [47]	88.9	30.6	57.3	86.1	88.9	30.6
Bryans 2011 [48]	69.4	41.7	74.0	80.6	69.4	41.7
Seffinger 2010 [49]	97.2	66.7	69.8	91.7	97.2	66.7
Deng 2009 [50]	58.3	69.4	51.0	97.2	58.3	69.4
Ravindran 2009 [51]	94.4	30.6	60.4	88.9	94.4	30.6
Filshie 2006 [52]	83.3	38.9	14.6	72.2	83.3	38.9
Suchowersky 2006 [53]	94.4	72.2	66.7	80.6	94.4	72.2
Anderson-Peacock 2005 [54]	97.2	47.2	77.1	77.8	97.2	47.2
Werneke 2005 [55]	69.4	50.0	27.1	69.4	69.4	50.0
Mechanick 2003 [56]	77.8	72.2	71.9	94.4	77.8	72.2

### Average appraisal scores, average overall assessments and recommendations regarding use of guidelines

Average appraisal scores, average overall assessments, and recommendation regarding use for each guideline are shown in Additional file 2. The average appraisal scores for each of the 17 guidelines ranged from 3.3 to 5.5 on the seven-point Likert scale (where seven equals strongly agree that the item is met); 14 guidelines achieved or exceeded an average appraisal score of 4.0, and seven guidelines achieved or exceeded an average appraisal score of 5.0. Average overall assessments for the 17 guidelines ranged between 3.0 (lowest) and 5.5 (highest), including 14 guidelines equalling or exceeding a score of 4.0, and 7 guidelines equalling or exceeding a score of 5.0.

### Overall recommendations (Table 2)

None of the 17 guidelines were recommended by both appraisers. Appraisers agreed in their overall recommendation for 13 of 17 guidelines including 2 No [52, 55], and 11 Yes with modifications [40–43, 47–49, 51, 53, 54, 56]. Of the remaining four guidelines, three were rated by the two appraisers as No and Yes with modifications [44, 45, 50], while 1 guideline was rated at Yes and Yes with modifications [46].

### Scaled domain percentage quality assessment (Table 3)

With regards to scaled domain percentages, scope and purpose scores were 52.8 to 100.0 %, stakeholder involvement scores were 11.1 to 86.1 %, rigor-of-development scores were 14.6 to 92.7 %, clarity-of-presentation scores ranged from 69.4 to 97.2 %, applicability scores were 0.00 to 60.42 %, and editorial independence scores ranged from 0.0 to 95.8 %.

### Scope and purpose

The overall objectives and health questions were generally well-defined in all but one guideline [45]. Authors provided the goal of the guideline, the types of CAM they sought to assess, and the disease or condition that was the target of CAM therapy or therapies. The population to whom the guideline was meant to apply was sometimes less detailed. For example, two guidelines referred to the intended population as “patients” [46, 48].

### Stakeholder involvement

Most guidelines thoroughly in detailed the characteristics of the members of the guideline development group, typically including degrees held by, and institutional affiliation of each member, in addition to some of the following: subject discipline, geographical location, and description of member’s role in the group [41–44, 46–49, 53–56]. Some guidelines detailed the views and preferences of the target population [44, 46, 53, 56] while most did not [40–43, 45, 47–52, 54, 55]. Target users of the guideline were typically inconsistently defined. Some guidelines offered clear descriptions, for example, type of practitioner, specialty [40, 46, 49, 50, 52, 53, 56], while other guidelines offered few details about target users [43, 45, 47, 50, 54, 55].

### Rigor of development

Systematic methods were almost always used to search for evidence and the criteria for selecting the evidence were almost always clearly described [40–44, 46–51, 53, 54, 56], with the exception of a few guidelines [45, 52, 55]. The strengths and limitations of the body of evidence were clearly described in all guidelines except for one [45]. The methods for formulating the recommendations varied; while some guidelines provided a fair amount of detail on how consensus was reached [40, 42, 46, 48, 49, 53, 54, 56], other guidelines provided minimal information if not none at all [40, 41, 45, 47, 51, 55]. All authors considered some health benefits, side effects, and/or risks in formulating their recommendations, with the exception of one [52]. Nearly all guidelines provided an explicit link between their recommendations and the supporting evidence with the exception of two guidelines in which this was inconsistent [49, 52]. While some guidelines explicitly stated that they were externally reviewed by experts prior to publication [41, 46, 54, 56], many did not [42–44, 47, 48, 52, 55]. Some guidelines failed to mention the purpose and intent for, or the methods employed for the external review [40, 45, 49–51, 53]. Most guidelines did not include a procedure for updating the guideline [42, 44, 45, 47, 48, 50–56] and, among those that did, one guideline provided a detailed methodology [46].

### Clarity of presentation

Generally, all guidelines offered specific and unambiguous recommendations. However, many typically lacked one or more of the following details: identification of the intent/purpose, relevant population, or caveats. All 17 guidelines scored highly in presenting different options for the management of the condition or health issue, thus contributing to this high scaled domain percentage [40–56]. Key recommendations were also generally very easily identifiable.

### Applicability

One guideline discussed facilitators and barriers to implementation of the recommendations [49]. Three guidelines included advice and/or tools to support implementation of the recommendations [49, 54, 56]. No guidelines addressed the resource implications of implementing the recommendations. Two guidelines provided monitoring and auditing criteria, while 14 guidelines contained little to no such information.

### Editorial independence

Guidelines varied in reporting of the funding source or competing interests of the members of the guideline development panel. Several guidelines that declared a funding source did not state whether funding source influenced the content of the guideline [41, 42, 48, 53, 54, 56].

No guidelines explicitly stated that no funding supported their development. Guidelines also varied in reporting of competing interests. Several guidelines did not address competing interests [45, 51, 52, 55, 56]. While remaining guidelines did so, two did not specify how potential competing interests were identified or considered, or how they may have influenced the guideline development process or issuing of recommendations [50, 53].

## Discussion

To identify credible, knowledge-based resources upon which patients and health care professionals can base discussions and decisions about use of CAM, the purpose of this research was to assess the quantity and quality of CAM guidelines. This study identified 17 guidelines (nine specific CAM therapy, eight mixed CAM therapies) published in 2003 or later that were relevant to a variety of conditions and diseases. Quality as assessed by the 23-item AGREE II instrument varied widely across guidelines overall and by domain; two guidelines scored 5.0 or higher in both average appraisal score and average overall assessment [46, 49], and three guidelines scored 3.5 or lower in both of these metrics [45, 52, 55] (1 = strongly disagree; 7 = strongly agree that criteria are met).

To our knowledge, no previous studies have assessed the quantity and quality of guidelines on CAM therapies. Thus, we believe that this is the first study to assess the credibility and nature of CAM guidelines. The findings are similar to those of guidelines on other clinical topics. In this study of CAM guidelines, the scaled domain percentages from highest to lowest were clarity of presentation (85.3 %), scope and purpose (83.3 %), rigour of development (61.2 %), editorial independence (60.1 %), stakeholder involvement (52.0 %) and applicability (20.7 %). In a previous study we found that, among 137 guidelines on a wide variety of clinical topics published from 2008 to 2013, the scaled domain percentages were ordered in similar fashion from highest (clarity of presentation 76.3 %) to lowest (applicability 43.6 %) [33]. Previous studies that examined a total of 654 guidelines published from 1980 to 2007 [57, 58], and 1,046 guidelines produced between 2005 and 2013 by 130 Australian guideline developers [59] also reported similar findings. Therefore the variable and sub-optimal quality of guidelines is not a unique phenomenon.

Notable strengths of this study included the use of a comprehensive systematic review to identify eligible CAM guidelines and the use of the validated AGREE II instrument by which to assess their quality, which is the internationally-accepted gold standard for appraising guidelines [38]. The interpretation of these findings may be limited by the fact that guidelines were independently assessed by two appraisers instead of four as recommended by the AGREE II instrument to optimize reliability. To mitigate this and standardize scoring, ARG, JYN and LL conducted an initial pilot-test during which they independently appraised the same two guidelines, then discussed the results and achieved consensus on how to apply the AGREE II instrument. Following appraisal of the 17 guidelines, ARG met with JYN and LL to discuss and resolve any uncertainties without unduly modifying legitimate discrepancies. This review does not address all CAM therapies; three therapies were chosen (herbal medicine, acupuncture, chiropractic or osteopathic manipulation) because they were identified as having the largest evidence base, and were therefore considered more likely to be the subject of guidelines [39]. We may not have identified all guidelines that included these three types of CAM therapy because, to establish a feasible scope, we did not search for guidelines on specific clinical topics and then peruse them for CAM-related content, and we did not search all CAM journals or the Guidelines International Network guideline library. We included CAM topics for which there was likely to be available evidence such as guidelines. Many patients use CAM lacking supporting evidence, therefore, it may be useful to examine guidelines on a broader range of CAM topics to evaluate the basis for recommending those therapies.

By describing the quantity and quality of CAM guidelines, this study revealed that few CAM guidelines are available to support informed and shared decision-making among patients and health care professionals. This likely reflects the lack of research on CAM therapies. Others have identified numerous factors that challenge CAM research including negative attitudes about CAM therapies [60–65] and a lack of targeted funding [66–69]. However, this is expected to change given that CAM therapies continue to be used by more than 40 % of the population in some regions of the world [70, 71]; and patients continue to use CAM despite documented risks associated with some CAM therapies [22, 70–75]. As research emerges, so too will guidelines that focus on CAM therapies [10].

This study also revealed that the quality of CAM guidelines varied across domains within individual guidelines, and across different guidelines. This finding is relevant to those who will produce CAM guidelines in the future, and to developers of existing CAM guidelines that, when updated, could be improved. Apart from the AGREE II instrument, numerous principles, frameworks, criteria and checklists are available to help guideline developers, including CAM guideline developers, to generate the highest-quality products [76–81].

## Conclusions

This study identified 17 guidelines published since 2003 on CAM therapies including herbal medicines, acupuncture, and chiropractic or osteopathic manipulation. Appraisal of these guidelines with the AGREE II instrument revealed that quality varied within and across guidelines. Some of these guidelines that achieved higher AGREE II scores and favourable overall recommendations could be used by patients and health care professionals as the basis for discussion about the use of these CAM therapies. In future updates, guidelines that achieved variable or lower scaled domain percentage and overall recommendations could be improved according to specifications in the AGREE II instrument, and with insight from a large number of resources that are available to support guideline development and implementation [75–80]. However, the fact that few CAM guidelines are available to support informed and shared decision-making between patients and health care professionals may continue to foster underuse of beneficial CAM therapies, and overuse or contraindicated use of other CAM for which there is no proven benefit or potential associated risks. This finding justifies the need for greater research on the safety and effectiveness of CAM therapies. Future research should also identify CAM therapies other than those reviewed here which are supported by sufficient evidence to serve as the basis for guideline development.

## Additional files

Additional file 1: MEDLINE Search Strategy for CAM guidelines executed Jan 28, 2016. (DOCX 33 kb)
Additional file 2: Average appraisal scores and average overall assessments of each guideline. (DOCX 37 kb)

## Abbreviations

AGREE II: Appraisal of guidelines for research & evaluation II
BMC CAM: BioMedCentral complementary and alternative medicine
CAM: Complementary and alternative medicine
JACM: Journal of complementary and alternative medicine
NCCIH: National Center for Complementary and Integrative Health
NGC: National Guideline Clearinghouse
PICO: Patients, intervention, comparison and outcomes
PRISMA: Preferred reporting items for systematic reviews and meta-analyses
