# Supplementary material for: The quantity and quality of complementary and alternative medicine clinical practice guidelines on herbal medicines, acupuncture and spinal manipulation: systematic review and assessment using AGREE II
Source: BMC Complement Altern Med. 2016 Oct 29;16:425. doi: 10.1186/s12906-016-1410-8 (PMC5086054; doi:10.1186/s12906-016-1410-8)
Supplement: Additional file 1: — MEDLINE Search Strategy for CAM guidelines executed Jan 28, 2016. (DOCX 33 kb) [file 12906_2016_1410_MOESM1_ESM.docx]

## Additional File 1: MEDLINE Search Strategy for CAM guidelines executed Jan 28, 2016

| 1 alternative medicine.mp. (7015)  2 alternative therap*.mp. (9266)  3 complementary medicine.mp. (2155)  4 complementary therap*.mp. (16209)  5 exp Complementary Therapies/ (188622)  6 (integrat* adj1 (medicine or therap*)).mp. [mp=title, abstract, original title, name of substance word, subject heading word, keyword heading word, protocol supplementary concept word, rare disease supplementary concept word, unique identifier] (2595)  7 exp Integrative Medicine/ (969)  8 CAM.mp. (20027)  9 naturopath*.mp. (1248)  10 exp Naturopathy/ (899)  11 acupunctur*.mp. (21450)  12 exp Acupuncture Analgesia/ or exp Acupuncture Points/ or exp Acupuncture Therapy/ or exp Electroacupuncture/ or exp Acupuncture/ (19327)  13 chiropract*.mp. (6037)  14 exp Chiropractic/ (3069)  15 (herb* adj1 (medic* or therap* or supplement*)).mp. [mp=title, abstract, original title, name of substance word, subject heading word, keyword heading word, protocol supplementary concept word, rare disease supplementary concept word, unique identifier] (15420)  16 exp Medicine, East Asian Traditional/ or exp Medicine, Chinese Traditional/ or exp Herbal Medicine/ or exp Plants, Medicinal/ or exp Phytotherapy/ (93506)  17 tcm.mp. (6179)  18 exp Drugs, Chinese Herbal/ (32295)  19 traditional Chinese medicine.mp. (10583)  20 exp Medicine, Ayurvedic/ (1886)  21 ayurved*.mp. (4225)  22 acupressure.mp. (887)  23 exp Acupressure/ (519)  24 applied kinesiolog*.mp. [mp=title, abstract, original title, name of substance word, subject heading word, keyword heading word, protocol supplementary concept word, rare disease supplementary concept word, unique identifier] (76)  25 exp Kinesiology, Applied/ (240)  26 herbalism.mp. [mp=title, abstract, original title, name of substance word, subject heading word, keyword heading word, protocol supplementary concept word, rare disease supplementary concept word, unique identifier] (119)  27 exp Osteopathic Medicine/ or exp Manipulation, Osteopathic/ (3442)  28 osteopath*.mp. (6313)  29 or/1-28 (306070)  30 limit 29 to ("all infant (birth to 23 months)" or "all child (0 to 18 years)" or "newborn infant (birth to 1 month)" or "infant (1 to 23 months)" or "preschool child (2 to 5 years)" or "child (6 to 12 years)" or "adolescent (13 to 18 years)") (32125)  31 29 not 30 (273945)  32 31 (273945)  33 limit 32 to (english language and humans and yr="2003 - 2016" and (guideline or practice guideline)) (113) |
| --- |

## 
