# Supplementary material for: The quantity and quality of complementary and alternative medicine clinical practice guidelines on herbal medicines, acupuncture and spinal manipulation: systematic review and assessment using AGREE II
Source: BMC Complement Altern Med. 2016 Oct 29;16:425. doi: 10.1186/s12906-016-1410-8 (PMC5086054; doi:10.1186/s12906-016-1410-8)
Supplement: Additional file 2: — Average appraisal scores and average overall assessments of each guideline. (DOCX 37 kb) [file 12906_2016_1410_MOESM2_ESM.docx]

## Additional File 2: Average appraisal scores and average overall assessments of each guideline

| Guideline | Metric | Appraiser 1 | Appraiser 2 | Average | Standard Deviation |
| --- | --- | --- | --- | --- | --- |
| Fogarty 2015 [40] | Appraisal Score | 4.6 | 4.9 | 4.8 | 0.2 |
|  | Overall Assessment | 4.0 | 4.0 | 4.0 | 0.0 |
| Bryans 2014 [41] | Appraisal Score | 4.7 | 4.7 | 4.7 | 0.1 |
|  | Overall Assessment | 5.0 | 5.0 | 5.0 | 0.0 |
| Greenlee 2014 [42] | Appraisal Score | 5.3 | 5.2 | 5.3 | 0.1 |
|  | Overall Assessment | 5.0 | 5.0 | 5.0 | 0.0 |
| Yadav 2014 [43] | Appraisal Score | 4.5 | 4.7 | 4.6 | 0.2 |
|  | Overall Assessment | 4.0 | 5.0 | 4.5 | 0.7 |
| Deng 2013 [44] | Appraisal Score | 4.2 | 5.3 | 4.7 | 0.7 |
|  | Overall Assessment | 4.0 | 5.0 | 4.5 | 0.7 |
| Liu 2013 [45] | Appraisal Score | 3.1 | 3.2 | 3.2 | 0.1 |
|  | Overall Assessment | 3.0 | 4.0 | 3.5 | 0.7 |
| Nahas 2013 [46] | Appraisal Score | 5.3 | 5.7 | 5.5 | 0.3 |
|  | Overall Assessment | 5.0 | 6.0 | 5.5 | 0.7 |
| Holland 2012 [47] | Appraisal Score | 4.3 | 4.4 | 4.4 | 0.0 |
|  | Overall Assessment | 4.0 | 4.0 | 4.0 | 0.0 |
| Bryans 2011 [48] | Appraisal Score | 4.7 | 4.5 | 4.6 | 0.1 |
|  | Overall Assessment | 5.0 | 5.0 | 5.0 | 0.0 |
| Seffinger 2010 [49] | Appraisal Score | 5.5 | 5.3 | 5.4 | 0.1 |
|  | Overall Assessment | 5.0 | 6.0 | 5.5 | 0.7 |
| Deng 2009 [50] | Appraisal Score | 4.2 | 4.3 | 4.3 | 0.1 |
|  | Overall Assessment | 4.0 | 4.0 | 4.0 | 0.0 |
| Ravindran 2009 [51] | Appraisal Score | 4.0 | 4.6 | 4.3 | 0.5 |
|  | Overall Assessment | 4.0 | 5.0 | 4.5 | 0.7 |
| Filshie 2006 [52] | Appraisal Score | 3.2 | 3.5 | 3.3 | 0.2 |
|  | Overall Assessment | 3.0 | 3.0 | 3.0 | 0.0 |
| Suchowersky 2006 [53] | Appraisal Score | 4.3 | 4.9 | 4.6 | 0.4 |
|  | Overall Assessment | 4.0 | 5.0 | 4.5 | 0.7 |
| Anderson-Peacock 2005 [54] | Appraisal Score | 5.2 | 5.5 | 5.4 | 0.2 |
|  | Overall Assessment | 5.0 | 5.0 | 5.0 | 0.0 |
| Werneke 2005 [55] | Appraisal Score | 3.1 | 3.4 | 3.3 | 0.2 |
|  | Overall Assessment | 3.0 | 3.0 | 3.0 | 0.0 |
| Mechanick 2003 [56] | Appraisal Score | 4.8 | 4.8 | 4.8 | 0.0 |
|  | Overall Assessment | 5.0 | 5.0 | 5.0 | 0.0 |
